# m^6^A transferase KIAA1429 mediates the upregulation of LncRNA LINC00968 promoting the progression of gastric cancer cells

**DOI:** 10.1186/s41065-025-00393-9

**Published:** 2025-03-11

**Authors:** Huijun Liu, Menghan Yang, Chunyue Zhang, Yanmin Zhang, Yan Wang, Yueda Chen

**Affiliations:** 1 School of Public Health, Puyang Medical College, Puyang, 457000 China; 2 Department of Hematology, The Second Affiliated Hospital of Anhui Medical University, Hefei, 230601 China; 3 Department of Oncology, Hengshui people’s Hospital, Hengshui, 053000 China; 4 Department of Neurosurgery, Hengshui people’s Hospital, Hengshui, 053000 China; 5 Department of General Surgery, Zhongshan Hospital (Xiamen), Fudan University, No. 668, Jinhu Road, Huli District, Xiamen, 361006 China; 6 Xiamen Clinical Research Center for Cancer Therapy, Xiamen, 361006 China

**Keywords:** lncRNA LINC00968, Gastric cancer, Cellular processes, N6-methyladenosine, KIAA1429

## Abstract

**Background:**

The screening and monitoring of gastric cancer is still a clinical challenge. Both N^6^-methyladenosine (m^6^A) and lncRNAs have been evidenced as critical regulators of gastric cancer, but their interaction and potential in modulating tumor progression remain unclear. This study aimed to evaluate the function of lncRNA LINC00968 in gastric cancer biological processes, and we discovered the role of KIAA1429, a typical m^6^A eraser, in mediating LINC00968 function.

**Materials and methods:**

The expression of LINC00968 was assessed using PCR and regulated by cell transfection. Cellular processes were evaluated by CCK8 and Transwell assays. The m^6^A modification and the interaction of LINC00968 with KIAA1429 were identified with Methylated RNA immunoprecipitation-qPCR. The regulatory effect of LINC00968 on miR-3202 and VIRMA was estimated by luciferase reporter assay.

**Results:**

Significantly increased LINC00968 was observed in gastric cancer cells. Silencing LINC00968 suppressed gastric cancer cell growth and motility. m^6^A-modified sites were predicted in LINC00968 and overexpressing KIAA1429 enhanced the enrichment and stability of LINC00968 in gastric cancer and reversed the knockdown of LINC00968. The overexpression of KIAA1429 could attenuate the inhibitory effect of LINC00968 knockdown on gastric cancer cellular processes. LINC00968 could negatively regulate the expression of miR-3202, which further regulate VIRMA, the coding gene of KIAA1429, in gastric cancer cells.

**Conclusions:**

LINC00968 contributes to the enhanced cell growth and metastasis of gastric cancer, which was mediated by KIAA1429-mediating m^6^A modification and the miR-3202/VIRMA axis.

**Supplementary Information:**

The online version contains supplementary material available at 10.1186/s41065-025-00393-9.

## Background

Gastric cancer dramatically threatened human health and lives, which ranked an important position in common malignant tumors and cause of cancer-related deaths. According to the latest data, it is estimated that the new cases and deaths of gastric cancer might increase to 26,890 and 10,880, respectively [1]. Great progress has been made in diagnostic methods and therapeutic strategies of gastric cancer, but the outcomes of patients remain unsatisfactory due to the unclear onset mechanism [2]. Surgery is the regular therapeutic strategy for early-stage gastric cancer, which could totally remove lesions, but is limited to patients at advanced stages. Adjuvant chemotherapy and radiotherapy have been developed for patients with metastasis, but recurrence and therapy resistance resulted in the adverse outcomes of patients [3]. Recent studies have proposed the potential of circulating tumor-sourced DNA, which could benefit the early detection of tumors, but it has not reached the level of clinical application. In the early stage of gastric cancer, there were no obvious symptoms increasing the difficulty of diagnosis and leading to missed diagnoses [4]. Environmental factors, infection of *Helicobacter pylori*, and host genes were all risk factors for gastric cancer, which involved a series of complicated pathological processes [5]. Molecules involved in these processes and their regulation would influence gastric cancer occurrence and development, which provides a number of candidates for gastric cancer biomarkers and has become hot point of recent research [6–8]. Meanwhile, gene mutation and dysregulation are also critical factors affecting tumor malignancy. Identification of molecular biomarkers could also provide potential therapeutic targets for cancer clinical management [9].

Although long non-coding RNAs (lncRNAs) are not involved in coding proteins, they have been demonstrated to regulate the cell cycle, cell differentiation, epigenetics, and various vital movements. Studies have evidenced that the dysregulation of lncRNAs is associated with their different function in the regulation of progression and drug resistance in various malignant tumors [10, 11]. Bioinformatic studies have also provided novel molecular insights into the progression of pan-cancer and explored a number of potential hub genes [12–15]. For example, lncRNA FOXD2-AS1 has been reported to promote cellular progression of OSCC and was considered a potential therapeutic target [16]. There have been several studies devoted to exploring dysregulated lncRNAs aiming to dig out as many potential biomarkers as possible, which dug out a series of candidate biomarkers [17–19]. lncRNA LINC00968 (LINC00968) was identified as a hub lncRNA related to the prognosis and ferroptosis of gastric cancer patients [20, 21]. LINC00968 has also been suggested to mediate tumor progression of several human cancer, such as lung adenocarcinoma, osteosarcoma, and ovarian cancer [22–24]. Whether LINC00968 could regulate the progression of gastric cancer remains unknown, which is critical for the identification of novel biomarkers.

N^6^-methyladenosine (m^6^A) is a common post-transcriptional modification, which affects several steps in gene expression, including RNA splicing, stabilization, translation, and output. m^6^A also widely existed in lncRNAs and varies from different cell and tissue types [25, 26]. m^6^A methylation is catalyzed by m^6^A writers, erasers, and readers, which consist of various enzymes and correlated proteins and display different functions. The role of m^6^A -related genes in cancer development has attracted special attention in recent studies [27, 28]. KIAA1429 was reported to regulate gastric cancer cell proliferation and chemotherapy sensitivity via modulating the stability of functional mRNAs [29–31]. The regulatory effect of KIAA1429 on lncRNAs and its involvement in the function of lncRNAs were indicated in a recent study on lncRNA LINC00958. LINC00958 was positively correlated with KIAA1429, which improves the stability of LINC00958 and mediates the promotion of gastric cancer aerobic glycolysis by LINC00958 in an m^6^A manner [32]. Therefore, m^6^A methylation mediated by KIAA1429 was speculated to be involved in the function of LINC00968 in gastric cancer. In this study, the function of LINC00968 in regulating cellular processes of gastric cancer was evaluated. The involvement and effect of KIAA1429 during the regulation of gastric cancer cells by LINC00968 were also assessed to confirm the role of m^6^A.

## Methods

### Association of LINC00968 with gastric cancer patients’ survival

Gene Expression Profiling Interactive Analysis (GEPIA) database (http://gepia.cancer-pku.cn/) could rapidly deliver customizable data based on The Cancer Genome Atlas (TCGA) and Genotype-Tissue Expression (GTEx) data. Therefore, it was employed to evaluate the significance of LINC00968 in the overall survival and disease-free survival of gastric cancer patients by searching the name “LINC00968”.

### Cell culture

MKN-45 and AGS cells and a normal human gastric epithelial cell (GES-1) were obtained from ATCC. Cell culture was conducted with the 10% FBS-containing DMEM culture medium at 37 °C. Cells with a confluence of 70–80% were available for the following experiments.

### Cell transfection

Cells were transfected with siRNA-LINC00968 or pcDNA 3.1 vectors cloned with KIAA1249 or LINC00968 cDNA, miR-3202 mimic (for the overexpression of miR-3202), or miR-3202 inhibitors (for the knockdown of miR-3202) for the regulation of LINC00968, KIAA1249, and miR-3202. Corresponding negative controls were also transfected using Lipofectamine 2000 (Invitrogen, USA) [33]. Efficiency was evaluated based on the expression of LINC00968 and miR-3202 using RT-qPCR. Cell transfection was performed at room temperature, and cells were collected after 48 h of transfection for the following analyses to reach a stable transfection efficiency and expression.

### Real-time quantitative PCR

Total RNA was extracted using RNeasy Mini kit (Qiagen, Germany) and assessed with NanoDrop 2000 (Thermo Scientific, USA). High-quality RNAs were reversely transcribed to cDNA using PrimeScript RT Master Mix (Takara, Japan) followed by PCR amplification on Step OnePlus Real-Time PCR System (Applied Biosystems, USA) with the following reaction conditions: 95 °C for 30 s followed by 40 cycles of 95 °C for 5 s and 60 °C for 30 s. The 2^−ΔΔCt^ method was used to calculate the relative expression with GAPDH as an internal reference [34]. The primer sequences are listed in Table S1.

### Cell proliferation assay

Suspension of cells (5 × 10^3^ cells/well) was seeded into 96-well plates from the left side of the hole near the bottom, and the edges were filled with sterile PBS solution. The plates were incubated in an incubator, and CCK8 reagent (10 µL/well) was added after 0, 24, 48, and 72 h of cell adhesion. The absorbance at 450 nm was detected after 2 h of CCK8 addition with a microplate reader (Molecular Device, USA) [35].

### Cell metastasis assay

The cell suspension was prepared in the FBS-free culture medium and seeded into the upper chamber 24-well Transwell plates [36]. The lower chamber was filled with 20% FBS-containing culture medium and make sure there are no air bubbles between the lower culture medium and the chamber. After incubating for 48 h, liquid in the upper chamber was removed and the metastatic cells were fixed with 4% polyformaldehyde for 30 min and stained with 1% crystal violet for 20 min at room temperature. The upper chamber was pre-coated with Matrigel (melted at 4 °C and diluted with an FBS-free culture medium) on the ice for invasion assessment. The chambers were incubated for 3–5 h after Matrigel coating and then were available for the experiments. The number of migrated and invasive cells were counted with an optic microscope and the relative migration and invasion by the ratio to the CK group (%).

### RNA stability assay

MKN-45 and AGS cells (5 × 10^5^ cells/well) were seeded into 6-well plates exposed to 2 µg Actinomycin D to inhibit the transcriptional processes of RNA polymerase, and DMSO was employed as control. Cells were collected after 0, 2, 4, and 6 h, and the stability of LINC00968 was evaluated by RT-qPCR according to the above description.

### m^6^A site prediction and MeRIP-qPCR

The m^6^A modification sites of LINC00968 were predicted with the online prediction tool SRAMP (http://www.cuilab.cn/sramp) by searching the sequence of full transcription. The parameters were set as default. The thresholds for very high/high/moderate/low confidence m^6^A sites correspond to the threshold achieved 99%, 95%, 90%, 85% specificities on cross-validation tests, respectively.

The immunoprecipitation buffer was prepared with Tris (pH 7.5), 1% NP-40, NaCl, and EDTA at 4 °C overnight. The protein A/G magnetic beads were conjugated with KIAA1429 antibodies (Abcam, USA) or IgG antibodies (as control) at room temperature for 30 min. RNA was incubated with the antibody with RNase and protease inhibitors. The precipitation RNA was analyzed by RT-qPCR after elution and purification. Total RNA extracted without immunoprecipitation was set as the Input. The relative enrichment was calculated by the ratio of RIP RNA/Input RNA [37].

### Prediction of LINC00968- and KIAA1429-related miRNAs

miRNAs correlated with LINC00968 and VIRMA, the coding gene of KIAA1429, were predicted with the following methods. The downstream miRNAs of LINC00968 were only predicted by lncBook (https://ngdc.cncb.ac.cn/lncbook) and lncRNASNP (http://gong_lab.hzau.edu.cn/lncRNASNP3/) database by searching the name “LINC00968” and transcript ID, respectively. While the upstream miRNAs of VIRMA was only enriched by miRDB (http://www.mirdb.org/mirdb/index.html) and miRWalk (http://mirwalk.umm.uni-heidelberg.de/) databases by searching the name “LINC00968”. The enriched results were taken intersection by Venn plotting using an online platform for data analysis and visualization (https://www.bioinformatics.com.cn)) [38].

### Statistical analyses

Data were presented as mean ± SD (*n* = 3) and analyzed with GraphPad Prism 9.0 software. Difference comparison was performed with a student’s t-test or one-way ANOVA (*P* < 0.05).

## Results

### Expression and function of LINC00968 in gastric cancer cells

**Fig. 1:**
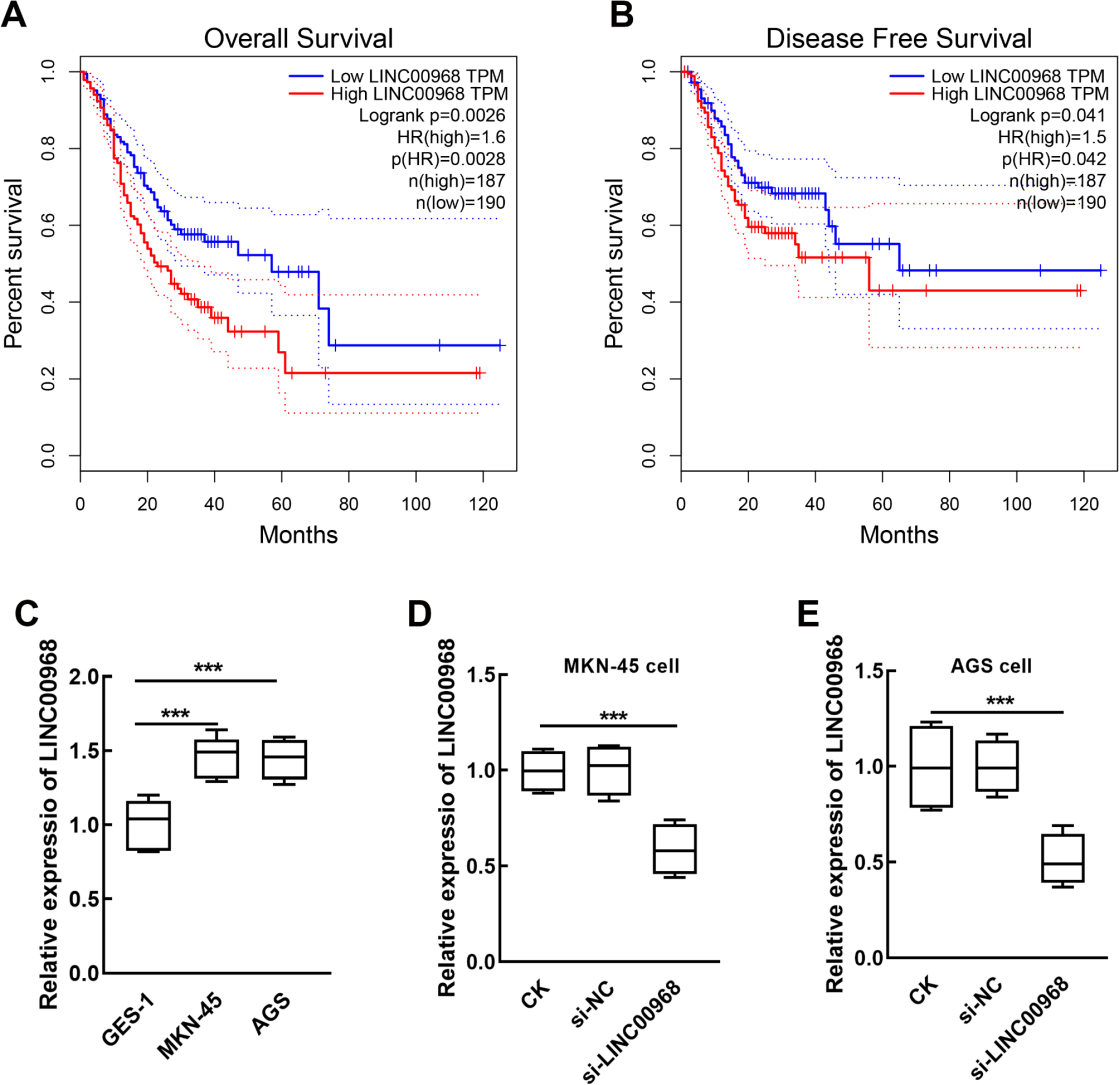
LINC00968 upregulation was significantly associated with lower overall survival (**A**) and disease-free survival (**B**) of gastric cancer patients (Data from GEPIA database). LINC00968 was significantly upregulated in gastric cancer cells, MKN-45 and AGS cells compared with a normal cell, GES-1 (**C**). The LINC00968-knockdown MKN-45 (**D**) and AGS (**E**) cells were established by cell transfection. ^***^P < 0.001 relatives to normal cell/CK. CK: blank control without any transfection; si-NC: small interference RNA negative control; si-LINC00968: LINC00968 small interference RNA for silencing LINC00968

According to the survival data from the GEPIA database, higher expression of LINC00968 was significantly associated with the overall survival rate (Fig. 1A) and disease-free survival rate (Fig. 1B) of gastric cancer, indicating its significant prognostic value in gastric cancer.

In gastric cancer cell lines, MKN-45 and AGS cells, LINC00968 expression was significantly higher than that in the GES-1 cell, a normal human gastric epithelial cell (*P* < 0.001, Fig. 1C). LINC00968 was significantly suppressed by the transfection of siRNA-LINC00968 in both MKN-45 (*P* < 0.001, Fig. 1D) and AGS cells (*P* < 0.001, Fig. 1E).

**Fig. 2:**
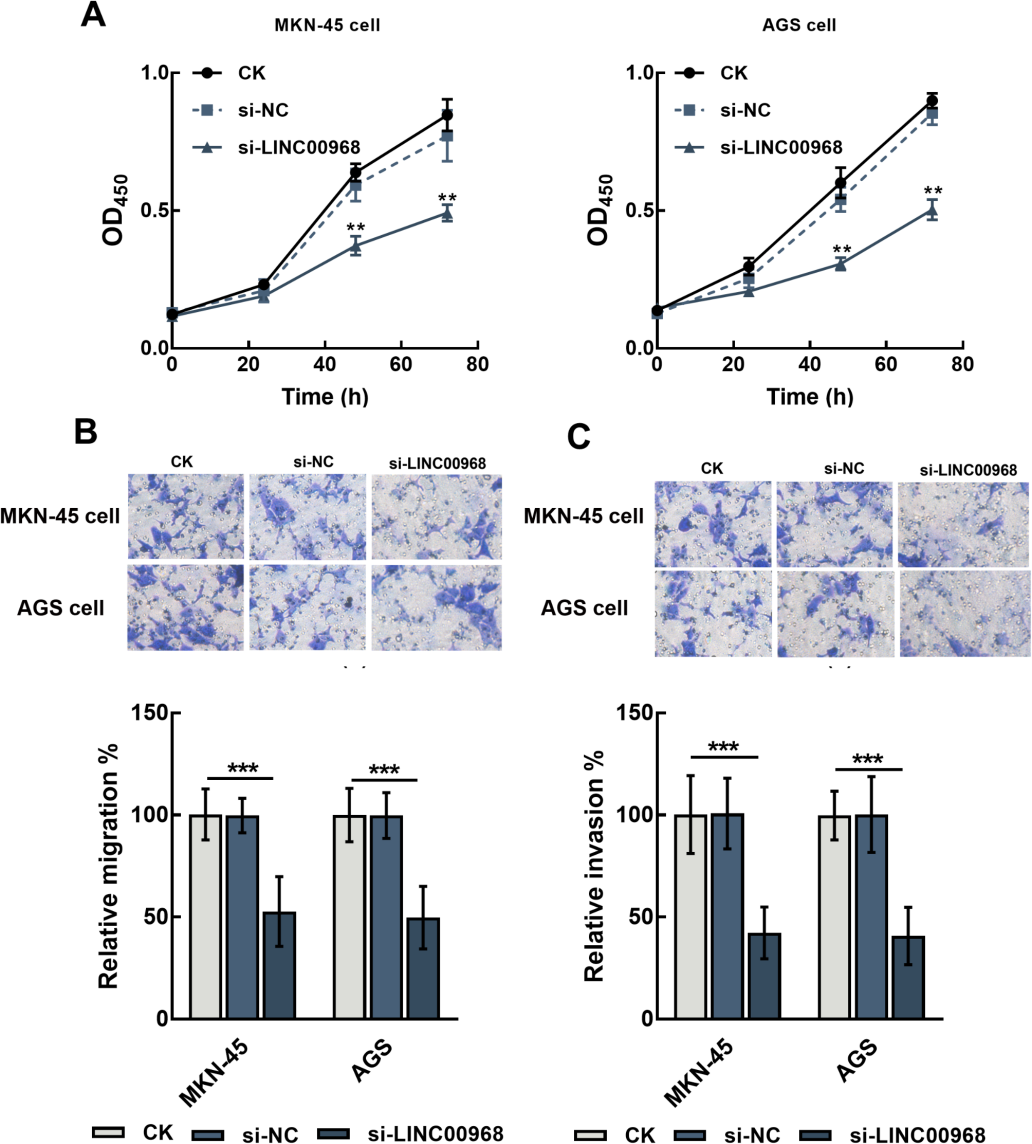
The effect of LINC00968 on gastric cancer cellular processes in MKN-45 and AGS cells. Silencing LINC00968 dramatically suppressed the proliferation (**A**), migration (**B**), and invasion (**C**) of MKN-45 and AGS cells. ^**^P < 0.01, ^***^P < 0.001 relative to CK. CK: blank control without any transfection; si-NC: small interference RNA negative control; si-LINC00968: LINC00968 small interference RNA for silencing LINC00968

Silencing LINC00968 significantly suppressed the proliferation of MKN-45 and AGS cells (*P* < 0.01, Fig. 2A). Meanwhile, the migration (*P* < 0.01, Fig. 2B) and invasion (*P* < 0.001, Fig. 2C) of MKN-45 and AGS cells were also inhibited by the knockdown of LINC00968.

### KIAA1429 mediated the modification of LINC00968 in m^6^A manner

**Fig. 3:**
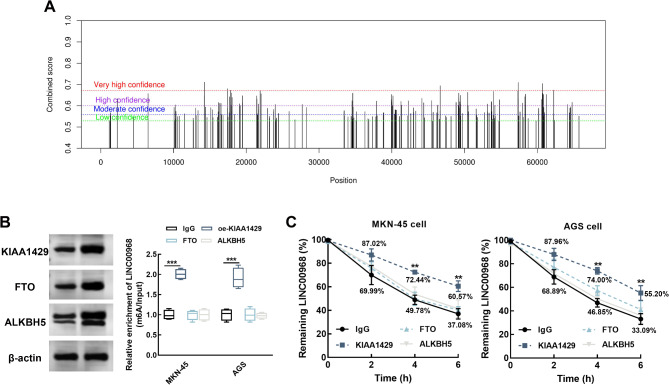
KIAA1429 is positively correlated with the enrichment and stability of LINC00968. (**A**) The m^6^A modification sites of LINC00968 predicted with the SRAMP (http://www.cuilab.cn/sramp). The combined scores indicate the bindary strength between LINC00968 and m^6^A enzymes, while the position indicate the potential m^6^A position in LINC00968. LINC00968 was enriched in KIAA1429-overexpressed MKN-45 and AGS cells compared with the control antibody, IgG (**B**). Overexpressing KIAA1429 significantly improved the stability of LINC00968 in MKN-45 and AGS cells. ^**^P < 0.01, ^***^P < 0.001 relatives to IgG antibodies (**C**)

With the help of an online predictor, a total of 204 m^6^A modification sites of LINC00968 was obtained, including 75 sites with high confidence, 66 sites with moderate confidence, and 63 sites with low confidence (Fig. 3A).

In MKN-45 and AGS cells, LINC00968 was dramatically enhanced by KIAA1429 antibodies compared with the control antibody, IgG, and other two m^6^A eraser antibodies, FTO and ALKBH5, indicating the combination of KIAA1429 and LINC00968 (*P* < 0.001, Fig. 3B). Additionally, the overexpression of KIAA1429 dramatically improved the stability of LINC00968 expression in gastric cancer cells, while IgG, FTO, and ALKBH5 showed no significant effects on it (*P* < 0.01, Fig. 3C).

**Fig. 4:**
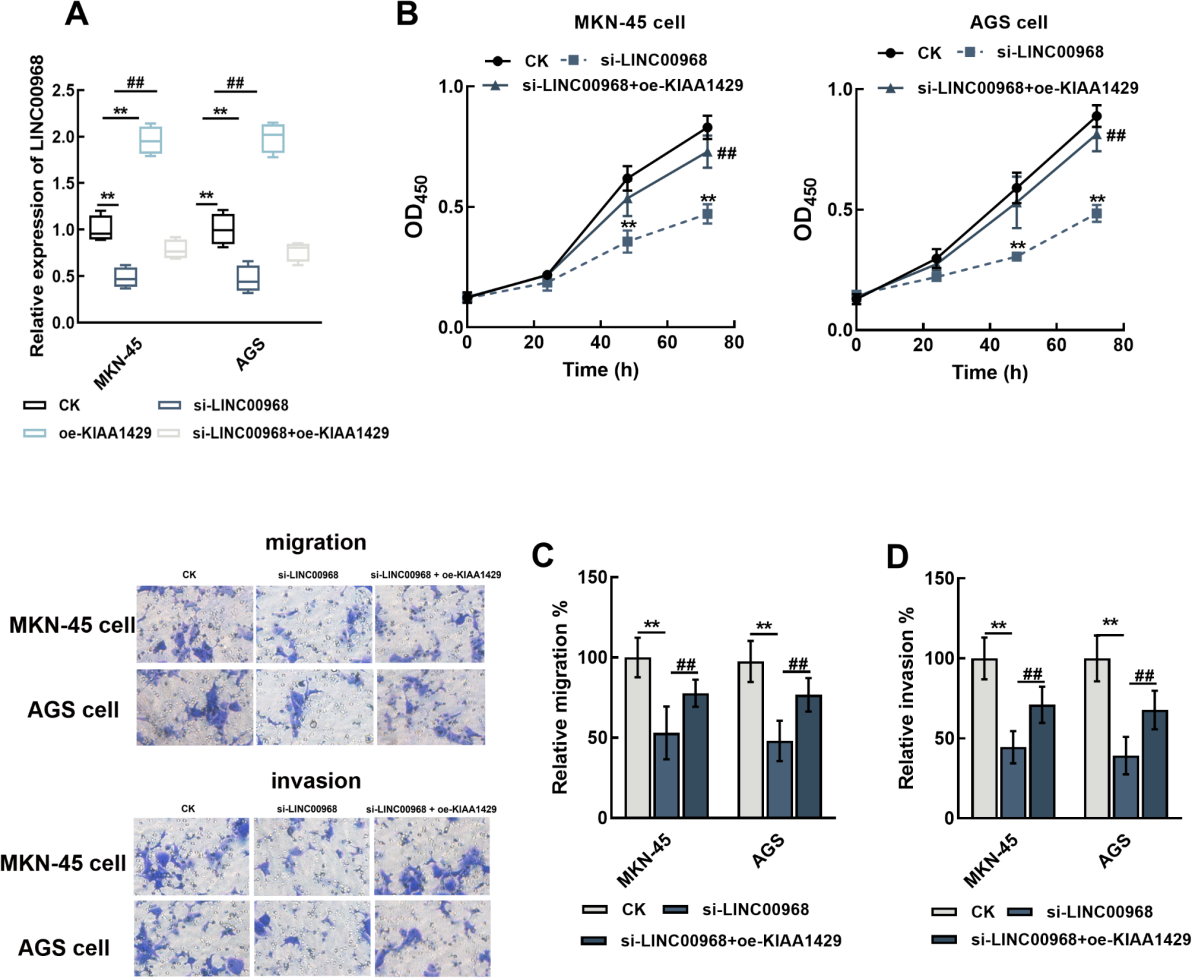
KIAA1429 mediated the function of LINC00968 in gastric cancer cells. Overexpressing KIAA1429 enhanced the expression of LINC00968 and reversed its knockdown (**A**). KIAA1429 overexpression could also alleviate the inhibitory effect of LINC00968 knockdown on proliferation (**B**), migration (**C**), and invasion (**D**) of MKN-45 and AGS cells. ^**^P < 0.01 relative to CK, ^##^*P* < 0.01 relative to si-LINC00968. CK: blank control without any transfection; si-NC: small interference RNA negative control; si-LINC00968: LINC00968 small interference RNA for silencing LINC00968; oe-KIAA1429: overexpressing KIAA1429

Overexpression of KIAA1429 significantly enhanced LINC00968 and could reverse the inhibitory effect of siRNA-LINC00968 in gastric cancer cells (*P* < 0.01, Fig. 4A). Moreover, overexpressing KIAA1429 could also alleviate the suppressed cell proliferation (*P* < 0.01, Fig. 4B), migration (*P* < 0.01, Fig. 4C), and invasion (*P* < 0.01, Fig. 4D) of gastric cancer by LINC00968 knockdown.

### miR-3202 mediated the interaction between LINC00968 and KIAA1429

**Fig. 5:**
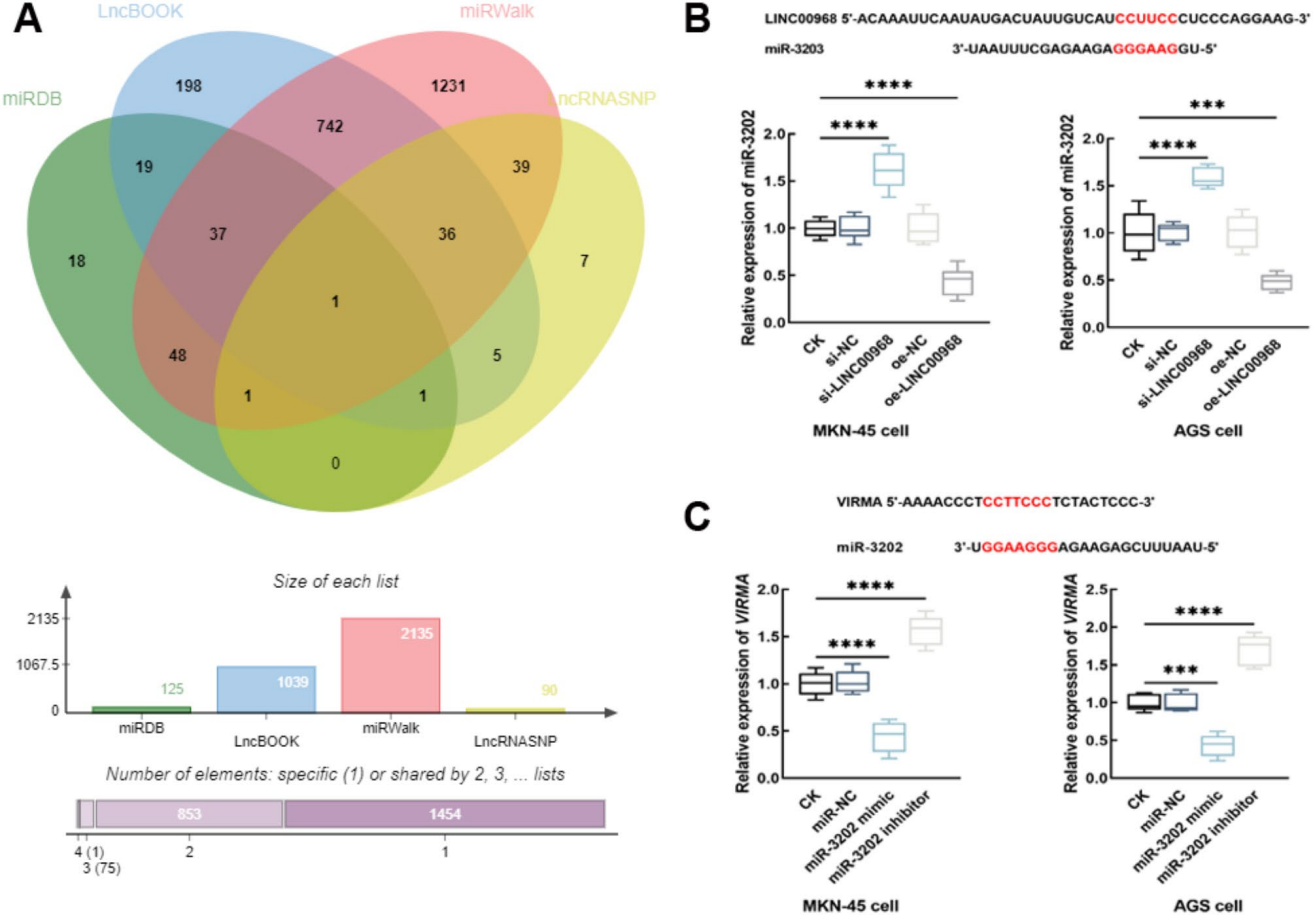
miR-3202 mediated the interaction between LINC00968 and KIAA1429. miR-3202 was enriched from the downstream miRNA of LINC00968 and the upstream miRNA of the KIAA1429-coded gene, VIRMA (**A**). LINC00968 negatively regulated the expression of miR-3202 in MKN-45 and AGS cells (**B**), and miR-3202 further negatively regulated the expression of VIRMA (**C**). ^***^P < 0.001, ^****^P < 0.0001. CK: blank control without any transfection; si-NC: small interference RNA negative control; si-LINC00968: LINC00968 small interference RNA for silencing LINC00968

The downstream miRNAs of LINC00968 were predicted by lncBook and lncRNASNP database, where a total of 1039 and 90 miRNAs were enriched, respectively. On the other hand, the upstream miRNAs of VIRMA were predicted from miRDB and miRWalk databases, and a total of 125 and 2135 miRNAs were obtained. While only miR-3202 was enriched in all four databases (Fig. 5A).

miR-3202 was predicted to bind with LINC00968 with several sites. In MKN-45 and AGS cells, the interaction between LINC00968 and miR-3202 was evaluated. It was found that the knockdown of LINC00968 significantly enhanced the expression of miR-3202, and the overexpression of LINC00968 showed the opposite effect (*P* < 0.0001, Fig. 5B). Additionally, miR-3202 could also bind with VIRMA at several sites, and it could also negatively regulate the expression of VIRMA in both MKN-45 and AGS cells (*P* < 0.0001, Fig. 5C).

## Discussion

The development of malignant tumors is a multiple-stage process involving complicated factors, where cellular processes play an important role. Recent studies have noticed the function of lncRNAs in mediating gastric cancer progression primarily through their regulatory effect on cell proliferation, apoptosis, metastasis, and other biological processes. For instance, upregulated lncRNA CASC11 in gastric cancer was demonstrated to facilitate cell proliferation, inhibit cell apoptosis, and block cell cycle and therefore promote the development of gastric cancer [39]. lncRNA EIF3J-DT could activate the autophagy of gastric cancer cells, which was associated with the chemoresistance of gastric cancer [40]. LINC00968 is located on chromosome 12q 12.1 and its abnormal expression and potential significance in the occurrence and development of gastric cancer were primarily discovered in recent studies, which primarily observed the potential of LINC00968 in ferroptosis and prognosis of gastric cancer [20, 21]. Meanwhile, LINC00968 also plays critical role in the development of various cancers. LINC00968 was downregulated in lung adenocarcinoma, which suppressed tumorigenesis and metastasis and inhibited cell growth [22, 41, 42]. The oncogene role of LINC00968 was indicated in osteosarcoma, where it showed significantly increased expression and induced enhanced cell migration and invasion [23]. Similarly, LINC00968 was also found to be upregulated in gastric cancer cells in the present study (Fig. 1C), and its knockdown dramatically suppressed cell growth and metastasis, suggesting its potential tumor promoter role in gastric cancer [20, 21].

m^6^A modification has attracted special attention in recent studies, and numerous investigations have identified m^6^A-related proteins as cancer biomarkers in malignant tumors, including but not limited to gastric cancer [43–45]. For example, YTHDF1, a typical m^6^A reader, was reported to promote the development of ovarian cancer [46]. The m^6^A methyltransferase METTL3 was also observed to act as a promoter in pancreatic cancer, which accelerated the proliferation and invasion of tumor cells [47]. In gastric cancer, m^6^A modification has also attracted huge attention [48]. For instance, the m^6^A modification of PKMYT1 by ALKBH5, a demethylase in gastric cancer was demonstrated to suppress cell invasion [49]. METTL16 was reported to promote cuproptosis in gastric cancer through the m^6^A modification of FDX1 [50]. Additionally, the m^6^A modification of lncRNAs has also been evidenced to mediate the development of human cancers. ALKBH5, as an m^6^A eraser, could inhibit the methylation of lncRNA NEAT1 and further facilitate metastasis and invasion of gastric cancer [51]. METTL14 was demonstrated to mediate the m^6^A modification and the promoted effect of upregulated LINC01320 on gastric cancer progression [37]. KIAA1429 plays a vital role in the methyltransferase complex and its significance in gastric cancer has also been revealed. In the present study, LINC00968 was also found to enrich in the presence of KIAA1429 overexpression. Moreover, KIAA1429 was also found to increase the stability and expression level of LINC00968 in gastric cancer cells. Additionally, overexpressing KIAA1429 could negatively regulate the knockdown of LINC00968 and alleviate the inhibitory effect of LINC00968 silencing on gastric cancer cell growth, migration, and invasion, suggesting the involvement of KIAA1429 in the tumor promoter role of LINC00968 in gastric cancer. Gastric cancer is closely related with the infection of *Helicobacter pylori*, which could promote tumor progression and affect patients’ outcomes [52]. Although there was no direct evidence demonstrating the association between m^6^A modification and *H. pylori* infection, the effect of *H. pylori* on m^6^A modification has been revealed in gastric cancer [53–55]. Hence, whether the m^6^A modification of LINC00968 by KIAA1429 was also influenced by the *H. pylori* is the future research direction of our study.

However, the position of m^6^A modification is also critical for the function of lncRNA [56]. The demethylation of the modified sites in the exon region would elevate the expression, and vice versa. The position of exon region might affect the nature of LINC00968, such as sense lncRNA or antisense lncRNA, whether this nature affect its function was unknown, which can be revealed in the future investigations. The modified sites in the cervical-loop structures or the binding positions of lncRNAs would significantly impact the stability of the transcript [57]. Although the m^6^A-modified sites of LINC00968 was predicted in this study, the regulatory sites of KIAA1429 have not been declaimed. Therefore, the m^6^A-modified sites of LINC00968 by KIAA1429 would help explain the mechanism underlying their interaction and function during the progression of gastric cancer. Additionally, the correlation between LINC00968 and KIAA1429 was significant from the data of the GEPIA database (Figure S1). Further studies would collect clinical samples to estimate the specific correlation and clinical significance of LINC00968 and KIAA1429 in the occurrence and development of gastric cancer. Animal modeling is also an effective way to deeply reveal the significance and function of LINC00968 in gastric cancer. Therefore, future studies would be devoted to constructing in vivo gastric cancer animal models to complete the role of LINC00968 in gastric cancer.

In mechanism, according to the prevalent competing endogenous RNA (ceRNA) theory, lncRNAs could serve as the sponge of miRNAs, and miRNAs would regulate the expression of target genes via binding with the 3’UTR, which mediate the function of lncRNAs. Here, LINC00968 was revealed to negatively regulate the expression of miR-3202, and miR-3202 also served as a negative regulator of VIRMA, the coding gene of KIAA1429. Therefore, LINC00968 was speculated to regulate the progression of gastric cancer cells via modulating the miR-3202/VIRMA axis and further regulating KIAA1429. Future investigations should focus on confirming the involvement of miR-3202 in the regulatory effect of LINC00968 on gastric cancer to complete the regulatory network and mechanisms.

## Conclusions

In conclusion, upregulated LINC00968 in gastric cancer served as a tumor promoter regulating cell growth and metastasis via modulating the miR-3202/VIRMA axis and regulating KIAA1429. Overexpressing KIAA1429 enhanced the expression of LINC00968 and mediated the effect of LINC00968 on gastric cancer cells in an m^6^A manner. These findings confirmed the involvement of miRNAs and highlighted the significance of m^6^A modification in the function of lncRNAs.

## Electronic supplementary material

Below is the link to the electronic supplementary material.

Supplementary Table S1: Primer sequences used in PCR

Supplementary Figure S1: Correlation between LINC00968 and KIAA1429 predicted from GEPIA database (http://gepia.cancer-pku.cn/index.html)

## Data Availability

The authors declare that they have no competing interests.
