# Supplementary material for: m6A transferase KIAA1429 mediates the upregulation of LncRNA LINC00968 promoting the progression of gastric cancer cells
Source: Hereditas. 2025 Mar 11;162:34. doi: 10.1186/s41065-025-00393-9 (PMC11895323; doi:10.1186/s41065-025-00393-9)
Supplement: Supplementary file 1 — Supplementary Table S1: Primer sequences used in PCR [file 41065_2025_393_MOESM1_ESM.docx]

Table S1. Primer sequences used in PCR

| Primer | Sequences |
| --- | --- |
| LINC00968 forward | 5'-GGCAGTTTTATTGTGGTGATT-3' |
| LINC00968 Revers | 5'-AATGGAAGTTGACGGGATAG-3' |
| miR-3202 forward | 5'-GCTGGAAGGGAGAAGAGC-3' |
| miR-3202 reverse | 5'-GTCGTATCCAGTGCAGGGTCCGAGGTAT  TCGCACTGGATACGACATTAAA-3' |
| VIRMA forward | 5'-AATCCTGTGGGAAGATCAGC-3' |
| VIRMA reverse | 5'-ACACGTAAGGCAGTGGTAAG-3' |
