# Supplementary figures and images for: m6A transferase KIAA1429 mediates the upregulation of LncRNA LINC00968 promoting the progression of gastric cancer cells
Source: Hereditas. 2025 Mar 11;162:34. doi: 10.1186/s41065-025-00393-9 (PMC11895323; doi:10.1186/s41065-025-00393-9)

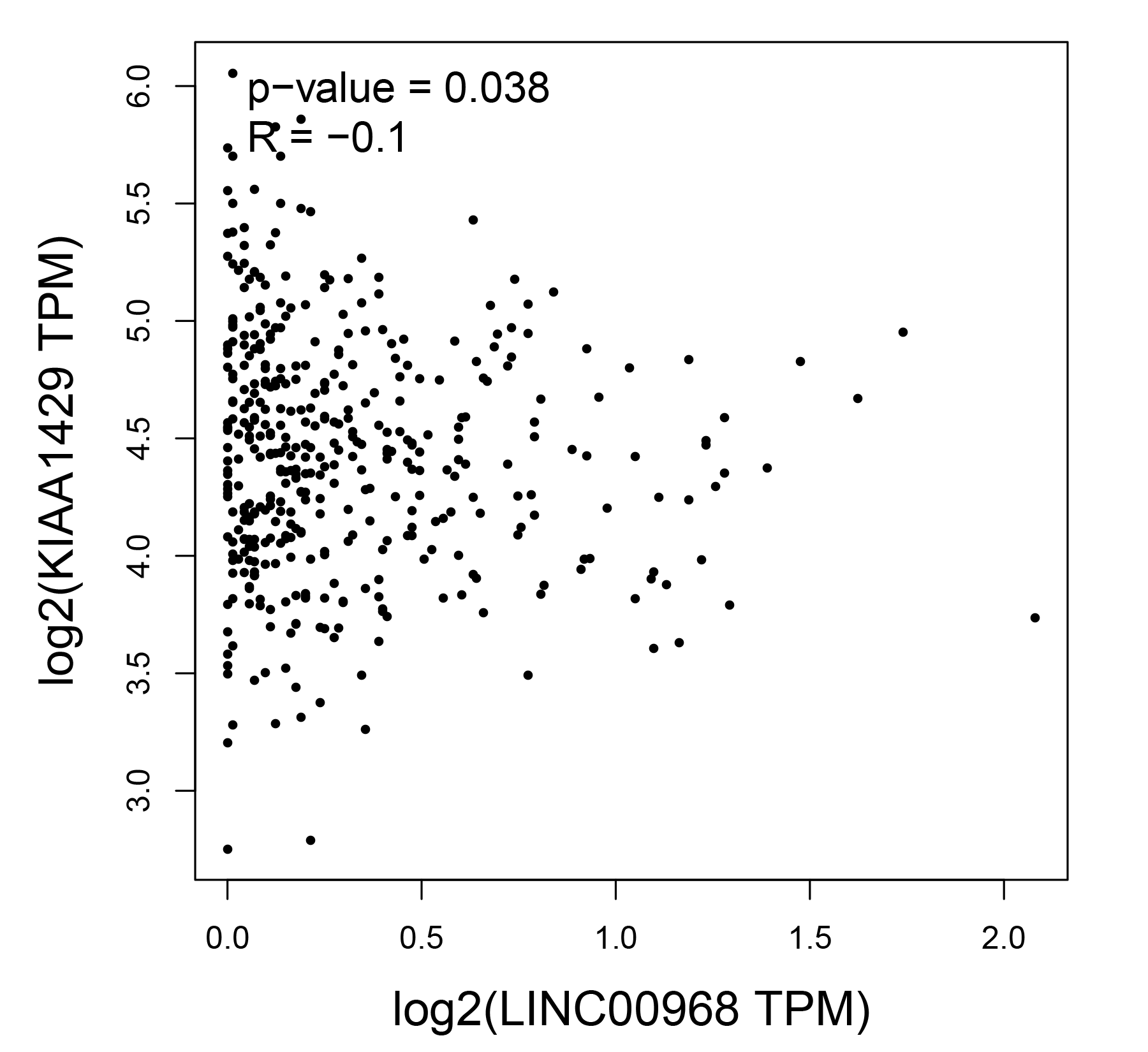

Supplement: Supplementary file 2 — Supplementary Figure S1: Correlation between LINC00968 and KIAA1429 predicted from GEPIA database (http://gepia.cancer-pku.cn/index.html) [file 41065_2025_393_MOESM2_ESM.png]
